# Cardiovascular magnetic resonance imaging as a gold standard for diagnosis of true aborted myocardial infarction

**DOI:** 10.1186/1532-429X-11-S1-P188

**Published:** 2009-01-28

**Authors:** Ingo Eitel, Georg Fuernau, Josef Friedenberger, Mahdi Sareban, Gerhard Schuler, Matthias Gutberlet, Holger Thiele

**Affiliations:** Heart Center Leipzig, Leipzig, Germany

**Keywords:** Myocardial Infarction, Percutaneous Coronary Intervention, Cardiovascular Magnetic Resonance, Primary Percutaneous Coronary Intervention, Delayed Enhancement

## Introduction

For detection and quantification of myocardial infarction, delayed enhancement cardiovascular magnetic resonance imaging (CMR) has emerged as the new gold standard. Furthermore, CMR has the unique advantage of visualization of even microinfarctions at very high spatial resolution.

Some patients who receive prompt reperfusion do not have a significant enzyme rise, but do exhibit typical ECG changes, which are consistent with an aborted myocardial infarction (MI). These patients are presumed that myocardial necrosis has been avoided. However, this has not been studied systematically with CMR.

## Purpose

The aim of this research was therefore to study CMR-parameters for the identification of aborted MI.

## Methods

To investigate the extent of aborted MI we examined 421 consecutive patients undergoing primary percutaneous coronary intervention (PCI) in acute STEMI within 12 h after symptom onset. Aborted MI was defined as maximal creatine kinase ≥2 upper limit of normal coupled with typical evolutionary electrocardiographic changes (ST-segment resolution > 50% within 2 h). All patients with aborted MI underwent CMR within 2–4 days using a 1.5 T MRI scanner. Left ventricular function was assessed by a standard steady-state free precession technique. For acute infarct determination, 3 short-axis slices using a T-2 weighted turbo spin-echo sequence were obtained. Early and delayed enhancement images covering the whole ventricle were acquired ≈1 and 15 minutes after intravenous administration of 0.15 mmol/kg body weighted gadobutrol (Gadovist, Schering, Germany) with an inversion recovery gradient echo sequence.

## Results

Of the 421 STEMI patients 53 patients (12.6%) fulfilled aborted MI criteria, with the highest frequency (22.4%) occuring in patients treated with primary PCI < 2 h after symptom onset.

In patients with aborted MI, CMR detected no delayed enhancement (DE) in 22 patients (41.5%), consistent with the absence of myocardial necrosis and infarction. In 31 (58.5%) patients CMR revealed transmural (27.3%) or non-transmural (31.2%) DE in the distribution of a coronary artery compatible with MI. As compared with true MI patients, patients with aborted MI had a significant lower infarct size (30.9 ± 19.6 ml vs. 8.5 ± 12.3 ml; 22.2 ± 12.7% vs. 5.9 ± 7.1%; p < 0.001 respectively) shorter pain-to-balloon time and a significant better left ventricular ejection fraction (46% vs. 60%, p < 0.001).

## Conclusion

CMR can distinguish between patients with true aborted MI with absence of myocardial scar and patients with fulfilled criteria of aborted MI but detected scar formation. Therefore CMR should be established as a diagnostic criteria for true aborted MI.

